# A phase I study of hydralazine to demethylate and reactivate the expression of tumor suppressor genes

**DOI:** 10.1186/1471-2407-5-44

**Published:** 2005-04-29

**Authors:** Pilar Zambrano, Blanca Segura-Pacheco, Enrique Perez-Cardenas, Lucely Cetina, Alma Revilla-Vazquez, Lucía Taja-Chayeb, Alma Chavez-Blanco, Enrique Angeles, Gustavo Cabrera, Karina Sandoval, Catalina Trejo-Becerril, Jose Chanona-Vilchis, Alfonso Duenas-González

**Affiliations:** 1 Unidad de Investigación Biomédica en Cáncer, Instituto Nacional de Cancerología/Instituto de Investigaciones Biomédicas, UNAM, Mexico; 2 Division of Clinical Research, Instituto Nacional de Cancerología, Mexico; 3 Laboratorio de Desarrollo de Métodos Analíticos, FES-Cuautitlán, UNAM, Mexico; 4 Laboratorio de Química Medicinal FES-Cuautitlán, UNAM, Mexico

## Abstract

**Background:**

The antihypertensive compound hydralazine is a known demethylating agent. This phase I study evaluated the tolerability and its effects upon DNA methylation and gene reactivation in patients with untreated cervical cancer.

**Methods:**

Hydralazine was administered to cohorts of 4 patients at the following dose levels: I) 50 mg/day, II) 75 mg/day, III) 100 mg/day and IV) 150 mg/day. Tumor biopsies and peripheral blood samples were taken the day before and after treatment. The genes APC, MGMT; ER, GSTP1, DAPK, RARβ, FHIT and p16 were evaluated pre and post-treatment for DNA promoter methylation and gene expression by MSP (Methylation-Specific PCR) and RT-PCR respectively in each of the tumor samples. Methylation of the imprinted H19 gene and the "normally methylated" sequence clone 1.2 was also analyzed. Global DNA methylation was analyzed by capillary electrophoresis and cytosine extension assay. Toxicity was evaluated using the NCI Common Toxicity Criteria.

**Results:**

Hydralazine was well tolerated. Toxicities were mild being the most common nausea, dizziness, fatigue, headache and palpitations. Overall, 70% of the pretreatment samples and all the patients had at least one methylated gene. Rates of demethylation at the different dose levels were as follows: 50 mg/day, 40%; 75 mg/day, 52%, 100 mg/day, 43%, and 150 mg/day, 32%. Gene expression analysis showed only 12 informative cases, of these 9 (75%) re-expressed the gene. There was neither change in the methylation status of H19 and clone 1.2 nor changes in global DNA methylation.

**Conclusion:**

Hydralazine at doses between 50 and 150 mg/day is well tolerated and effective to demethylate and reactivate the expression of tumor suppressor genes without affecting global DNA methylation

## Background

Cancer is considered to be a disease of the genome that results from a plethora of genetic and epigenetic lesions. Among the epigenetic alterations, DNA hypermethylation is thought to play an important role in tumor development and progression [1]. In this regard, at least three functional DNA methyltransferases (DNMTs) have been identified, the most abundant is DNMT1 which preferentially methylates hemi-methylated DNA [2], and plays a key role in imprinting and X-chromosome inactivation during embryogenesis [3,4]. DNTM1 localizes to replication foci [5], at least in part by interacting with proliferating cell nuclear antigen (PCNA), a protein closely involved in DNA replication. It is therefore responsible for maintaining proper methylation levels during replication and possibly repair [6]. Other known functional methyltransferases are DNMT3a and DNMT3b, which are responsible for *de *novo methylation during embryogenesis [7]. DNMT3a and DNMT3b have equal preferences for hemi-methylated and non-methylated DNA, and so have been classified as *de novo *methyltransferases [8]. DNA methylation can directly interfere with transcriptional factor binding and thus inhibit replication [9], with methyl-CpG binding proteins which bind methylated DNA and with regulatory proteins that inhibit transcription [10]. In addition, both DNMT1 and methyl-binding proteins (MBP), such as methyl-CpG-binding protein 2 (MeCP2) recruit histone deacetylases which deacetilate histone core tails leading to tighter chromatin packaging, reducing the access of transcriptional factors to DNA [11,12].

Cancer cells are considered to have global hypomethylation and regional hypermethylation. Hypermethylated regions are CpG islands, CpG and GpC rich sequences 1 kb long found proximal to gene promoters involved in transcriptional control [13]. These islands are associated with roughly half of all genes [15], their methylation can repress transcription in a manner analogous to a mutation or deletion (16). It is thought that tumor suppressor gene promoter hypermethylation contributes to their transcriptional silencing [14]. Furthermore, there is a growing list of tumor suppressor genes in both sporadic and familial cancers which are found to be transcriptionally silenced by hypermethylation [17].

In this regard, tumor suppressor gene transcriptional reactivation through promoter de-methylation represents an attractive strategy for anticancer treatment. Substantial preclinical studies characterizing DNA methylation inhibitors have shown cancer cell line growth arrest in vitro and antitumor effects in animal models, including survival prolongation [18-20]. These concepts are supported by the transforming effect of exogenous DNA methyltransferase gene expression observed in fibroblasts [21] as well as by the malignant phenotype reversion documented using antisense oligonucleotides against this gene [22]. These findings have paved the way for the clinical testing of demethylating agents in cancer.

Nucleoside deoxycytidine analogs formerly known as classic cytotoxic agents and later known as DNA methylation inhibitors show poor activity against solid tumors [23] however, 5-aza-2'-deoxycytidine has recently gained considerable attention and is presently being tested as a demethylating agent for the treatment of hematological neoplasms [24]. MG98, is an antisense oligodeoxynucleotide directed against the 3' untranslated region of the DNA methyltransferase-1 enzyme mRNA that has been tested in clinic [23]. A phase I study using biweekly administration of this agent, showed no consistent decrease of mRNA levels in the peripheral blood cells of patient [25]. Although this agent has shown activity in xenografts models of nude mice, demonstration of antitumor efficacy in humans is pending.

Our group has recently shown in vitro and in vivo promoter demethylation and tumor suppressor gene transcriptional reactivation mediated by the antihypertensive compound hydralazine [26]. Its DNA demethylating activity can be explained by the interaction between its Nitrogen atoms with residues Lys162 and Arg240 of the DNA methyltransferase active site as showed in a silico model [27]. Hydralazine is a well-tolerated drug devoid of the common side effects of cytotoxic chemotherapy agents, however, its hypotensive effects could limit its use in a clinical setting, we thus felt desirable to determine the dose at which its demethylating activities were observed in a set of genes known to be methylated in cervical cancer.

## Methods

### Patient selection

Previously untreated patients with histological diagnosis of carcinoma of the cervix were entered into this phase I study. The following inclusion criteria were applied: 1) age between 18 and 75 years; 2) Karnofsky status 70% or higher; 3) hematological, renal and hepatic functions as follows: hematological: Hemoglobin equal or higher than 10 g/L, leukocytes >4000/mm^3^, platelets >100 000/mm^3^, total bilirubin and transaminases <1.5× the normal upper limit, and normal serum creatinine; 4) a normal chest X-ray and 5) signed informed consent. Exclusion criteria were: 1) history of allergy to hydralazine; 2) any past or current cardiovascular condition (higher blood pressure, heart failure, etc., that required pharmacological treatment; 3) any past or current rheumatic or autoimmune disease; 4) uncontrolled infection or other systemic diseases; 5) concomitant treatment with any experimental drug; 6) pregnant or nursing women; 7) mental illness; and 8) previous or concomitant malignant diseases other than non-melanoma skin cancer. The Institutional Regulatory Board approved the study protocol.

### Clinical samples and nucleic acids extraction

Biopsies were taken from areas with visible macroscopic cervical tumor using a sterile biopsy punch the day before and the day after the 10 days of hydralazine treatment. The specimen was immediately frozen at -20°C for further processing. In addition, a blood sample of 10 mL was drawn from the arm by venipuncture for subsequent mononuclear cell DNA extraction. In addition, the surgical specimen of an early stage cervical cancer patient undergoing radical hysterectomy was multi-sampled in the surgical room by taking 4 small fragments of the macroscopic tumor from separate areas. Genomic DNA was isolated from the tumor tissues and from the buffy coat layer of blood samples using the standard method of proteinase K digestion and phenol-chloroform extraction. RNA from tumor biopsies was obtained using the TriReagent (Gibco BRL Grand Island, New York) RNA extraction kit following the manufacturer instructions.

### Hydralazine treatment

Twenty four hours after tumor and blood sampling patients where divided into the following groups and started on oral hydralazine for a ten day period: I) 25 mg every 12 hours, II) 25 mg every 8 hours; III) 50 mg every 12 hours; and IV) 50 mg every 8 hours. Toxicity was assessed at the end of study period (10 days), with special emphasis on the presence, throughout the treatment days, of known signs and symptoms associated with hydralazine treatment (hypotension, tachycardia, palpitations, syncope, sweating, headache, dizziness and fluid retention). The study period ended at day ten and patients went to receive definitive treatment.

### Analysis of DNA methylation in the tumor

The methylation status of genes was determined in the biopsies pre and post-treatment. The gene set studied was: p16, RARβ, MGMT, ER, FHIT, APC, DAPK and GSTp1 which were analyzed by methylation-specific PCR as previously described [28]. In addition, the four fragments of the tumor of the untreated patient were analyzed for methylation of the DAPK gene. Briefly, 1 μg of DNA in a volume of 100 μl of each sample was denaturated with freshly prepared NaOH at a final concentration of 0.2 M, and modified according to the manufacturer instructions of the DNA Modification kit (Intergen, Purchase, New York). The PCR mixture contained 2 μl of 10X PCR buffer, 0.5 U of Taq Gold polymerase, dNTPs (each 1.25 mM), 300 ng of primers and bisulfite-modified DNA in a final volume 20 μl. Products were visualized in a 2% agarose gel under UV light. The primers and conditions for PCR are shown in Table 1. A gene was deemed methylated whenever a band was present after amplification with the methylated, or both methylated and unmethylated set of primers. On the contrary, a gene was deemed unmethylated when a band was present after amplification with the unmethylated set in the absense of band with the methylated set. An exception to these criteria was when in the pretreatment biopsy there was only a methylated band but both unmethylated and methylated bands after treatment.

### Analysis of DNA methylation in mononuclear cells of peripheral blood

The methylation status of the imprinted H19 gene as well as the clone 1.2 which is known to be normally methylated and not subjected to imprinting [29] was also analyzed by methylation specific PCR. The positive control for unmethylation in the clone 1.2 sequence was DNA from cultured cells treated with ethionine. Primers and conditions are shown in Table 1.

### Analysis of gene expression

Total RNA obtained from pre and post-treatment biopsies was reverse transcribed using a RT-PCR kit (Perkin Elmer; Branchburg, New Jersey) following the manufacturer instructions. Primers and conditions for amplifications are shown in Table 2.

### Quantification genomic 5-methylcytosine DNA content by capillary electrophoresis

Adenine, thymine, guanine, uracyl, cytosine and 5-methyl-cytosine were purchased from Sigma (St. Louis, MO; USA). Bases were dissolved at 2.4 mM in 0.1 M HCL, 0.01 M HCL, or Milli-Q-grade water (Millipore, Bedford, MA) and filtered through 0.45 μm pore size filters (Millipore). Briefly, extracted DNA samples were resuspended in TE buffer at (1 μg/μl). Hydrolysis of DNA was carried out by incubating 40 μg of DNA in 2 mL 88% v/v formic acid at 140°C into a sealed ampoule for 90 min. After hydrolysis, samples were reduced to dryness by speedvac concentration and redissolved in 30 μL of Milli-Q-grade water. DNA samples of pre and post-treatment biopsies were analyzed for 5-methylcytosine content by capillary electrophoresis (CE) as reported by us [30]. For the CE procedure, an uncoated fused-silica capillary (Beckman-Coulter; 60 cm × 75 μm; effective length, 44.5 cm) was used in a CE system (P/ACE MDQ; Beckman-Coulter) connected to a data-processing station (32 Karat software). The running buffer was 20 mM NaCO_3 _(pH 9.6 ± 1) containing 80 mM SDS. Running conditions were 25°C with an operating voltage of 20 kV. On-column absorbance was monitored at 223 nm. Before each run, the capillary system was conditioned by washing with the running buffer for 2 min. Buffers and washing solutions were prepared with Milli-Q water and filtered throughout 0.45-μm filters. Hydrolyzed samples, previously filtered through 0.45 μm pore filters, were injected under pressure (0.5 p.s.i.) for 15s. The relative methylation of each DNA sample was taken as the percentage of ^m^C in total cytosine: ^m^C peak area × 100/(C peak area + ^m^C peak area).

### Methylation pattern in global DNA

Global DNA methylation was evaluated by the cytosine-extension assay [31] which is based on the use of methylation-sensitive restriction endonuclease that leave a 5' guanine overhang after DNA cleavage, with subsequent single nucleotide extension with radiolabeled [(3)H]dCTP. Briefly 1 μg of DNA from tumor tissue was digested overnight with BssHII according to the manufacturer. Single nucleotide extension reaction was performed in a 25 μl reaction mixture containing 0.25 μg of DNA, 1X buffer II, 1 mM MgCl, 0.25 U of DNA polymerase (Perkin Elmer; Branchburg, New Jersey [^3^H]dCTP (Ci/mmol) and incubated at 56°C for 1 hour, then placed on ice. The reaction mixture was then applied to sephadex G25 column. For the column chromatography, each sephadex G25 column was centrifuged for 10 sec., at 5000 rpm to remove the buffer and loaded with the reaction mixture. After loading the samples to the column, the radiolabeled DNA was collected by centrifugation of the column and mixed with liquid scintillation for the determination of radioactivity. Results were expressed as the percentage change from the average controls which were tumor samples of patients pre and post-treatment without BssHII digestion.

## Results

### Study group

A total of 16 patients were studied. All of them were chemotherapy or radiation naive and had a macroscopic tumor accessible for punch biopsy. Their mean age was 51.8 (35–75 years), all cases were squamous histology and staged as FIGO stage IIB and IIIB. The status performance was 0–1 in all patients (Table 3).

### Treatment compliance and side effects

All patients completed the prescribed medication and reported that hydralazine treatment was well tolerated. Side effects as evaluated by the Common Toxicity Criteria are showed in Table 4. Grade 1 fatigue, headache and palpitations were observed in 50% of patients; grade 1 nausea was present in 37% of cases, being grade 2 in only one patient. Only 2 out of 16 patients presented dizziness grades 1 and 2 respectively. No patient stopped treatment due to adverse effects. We observed no side-effect dose relationship.

### Gene promoter methylation

**Figure 1:**
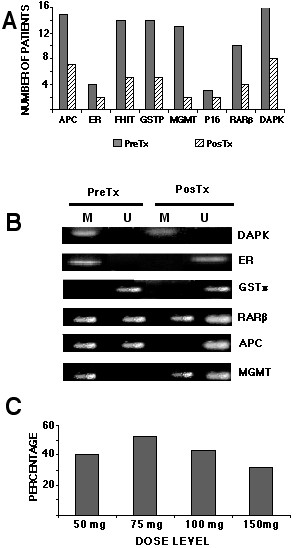
**1A**. Pre- (dark bars) and post-hydralazine treatment (light bars). The bars represent the number of patients that showed methylation for each studied gene from each of the 16 patients. **1B**. Representative cases of genes (M methylated, U unmethylated; pre/post): M/M; M/U; U/U, M/U; MU/U; M/ U-M. **1C**. Percentage of demethylation after treatment according to the dose. Percentage was calculated considering 100% methylation the total number of pre-treatment methylated genes in each cohort of 4 patients

Methylation analysis of the biopsies taken before and after 10 days of hydralazine administration was performed in all 16 patients. Methylation results involving the genes analyzed were variable. Overall, 70% (89 out of 128) of the pretreatment samples analyzed (8 genes for each of the 16 pre-treatment biopsies) had at least one methylated gene, and all 16 patients had at least one methylated gene. Analysis by individual genes showed the following rates of methylated genes: APC (94%), ER (25%) FHIT (88%), GSTp1 (88%), MGMT(81%), p16(19%), RARβ (62%), and DAPK (100%), irrespective of the dose of hydralazine used, the post-treatment biopsies showed a variable demethylation rate according to the gene, varying from 15% (2/13 samples) for the MGMT gene, to 67% of demethylation for the p16 gene (2 out of 3 samples), (Figure 1A). Representative cases of the results are shown in Figure 1B. Correlation between demethylation analysis and dose level revealed the following results: at 50 mg a day, 40% of methylated genes suffered demethylation; at 75 mg it was 52%, at 100 mg dose the rate was 43%, and 32% for the 150 mg dose (Figure 1C).

### Gene expression

**Figure 2:**
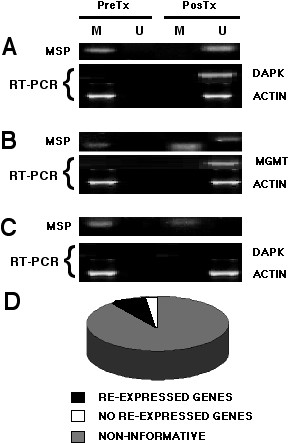
Representative cases correlating methylation and re-expression before and after hydralazine treatment. **2A **is a patient treated with 75 mg/day that demethylated and re-expressed the DAPK gene. **2B **corresponds to a patient receiving 150 mg/day who showed only the methylated band pre-treatment, but both bands after treatment, which correlated with re-expression of MGMT. **2C **is a 50 mg/day patient which failed to demethylate the DAPK gene and therefore lacked expression. **2D **represents the distribution of informative cases. From the 128 genes/cases, 116 were RT-PCR positive regardless of the methylation status, hence were not informative. In the remaining 12 cases, nine demethylated and re-expressed the gene.

All cases showed expression of β actin. Gene expression analysis showed that 90% (116 out of 128) of the tumor samples expressed the messenger in the pre-treatment and post-treatment biopsies regardless of methylation status, hence were not informative. On the other hand, there were only 12 informative cases. Of these 12 cases, three (25%) (having only the methylated band pretreatment) showed no re-expression after treatment (2/ DAPK, 1/GSTp1 cases). These three cases received the 50 mg dose. In the remaining 9 cases (75%) expression of the gene was re-induced after treatment. These nine cases behaved as follows: Five cases were RT-PCR negative pretreatment (only methylated band) and converted positive post-treatment, displaying methylated and unmethylated bands (FHIT two cases -100 and 150 mg doses- MGMT two cases -75 and 150 mg doses, GSTp1 one case -50 mg dose). Three cases RT-PCR negative pre-treatment with methylated band which converted to unmethylated and expression positive in the post-therapy biopsy (DAPK and ER genes at 75 mg, GSTp1 at 150 mg dose), and finally, a RT-PCR negative in the pre-treatment biopsy despite having methylated and unmethylated bands which converted to RT-PCR positive accompanied by only unmethylated band (GSTp1 case at 75 mg). Table 5, Figure 2D. Three representative cases are shown in Figure 2A, B, C.

### Methylation heterogeneity in tumor samples

**Figure 3:**
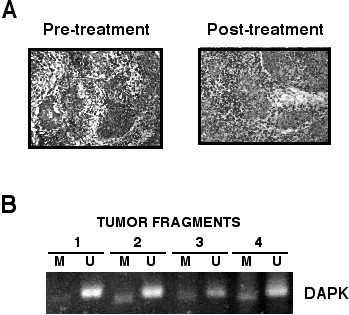
**3A**. Photomicrography of a representative set of pre and post-treatment tumor biopsies showing that the malignant component represents almost the half of the tumor. **3B**. Methylation analysis of the DAPK gene in the four fragments of the tumor biopsy of an untreated cervical cancer patient. Despite all fragments contained different proportions of malignant cells and stroma the four samples show methylated and unmethylated bands.

Because the sampling of tumor may lead to tissue specimens with different degree of "contamination" with non-malignant that may affect the result of methylation in the post-treatment, biopsies, all samples were analyzed by a pathologist. The results showed that in all biopsy samples the content of malignant cells varied from 30 to 70%. A representative set is shown in Figure 3A. In addition, we reasoned that if tumor heterogeneity were of a magnitude to yield different methylation patterns in the tumors regardless of the treatment, then multisampling a tumor with subsequent methylation analysis of each of the tumor fragments will render zones with different methylation pattern. The analysis of the surgical specimen of an untreated patient showed that the methylation of the DAPK gene promoter was the same in the four fragments of the tumor. Figure 3B.

### Methylation of imprinted and "normally methylated" genes

**Figure 4:**
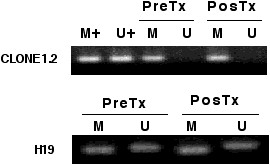
DNA methylation analysis obtained from peripheral mononuclear cells of genes "imprinted or normally methylated". Clone 1.2 remained methylated in all cases whereas for the imprinted H19 gene, the pattern of U and M alleles did not change.

The methylation status of the imprinted gene H19 was investigated in the DNA extracted from peripheral blood cells. As shown in Figure 4, no change in the expected pattern of bands was observed after treatment, all patients showed bands with the methylated and unmethylated set of primers. A consistent pattern of methylation also was observed in all patients analyzed for the "normally methylated" sequence *clone 1.2*. No case showed demethylation at this locus.

### Global methylation

**Figure 5:**
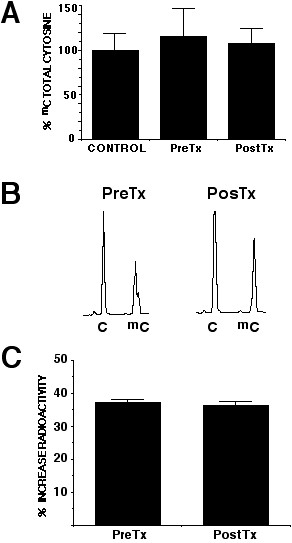
**5A**. Capillary electrophoretic analysis of global methylation. Relative methylation showed no variation in percentage of ^m^C after treatment (37.3% versus 36.3%). **5B **is a electropherogram showing the separation of C and ^m^C. **5C **is the percent increase in radiolabeled incorporation pre and post-treatment as compared to the control of undigested DNA.

Global tumor DNA methylation was evaluated by two methods, capillary electrophoresis and cytosine extension. Enough DNA to perform capillary electrophoresis was only available in five cases (four patients taking 100 mg, and one taking 150 mg/day respectively). The relative methylation of each DNA sample was taken as the percentage of d^m^C in total cytosine. Figure 5A shows the relative methylation in theses cases. Remarkably, relative methylation in all cases was between 34.2 and 38.4% and there was no change in methylation levels after hydralazine treatment (37.3% ± 0.81 and 36.3% ± 1.1 pre and post-treatment respectively). An electropherogram showing the separation of the analytes of interest, C and ^m^C peak are shown in Figure 5B. In the cytosine extension assay, the extent of [(3)H]dCTP incorporation after restriction enzyme treatment is directly proportional to the number of unmethylated (cleaved) CpG sites. The results of this assay showed no statistically significant changes in the tumor samples pre and post-treatment as the percentage increase in radiolabel incorporation were 15% ± 31.3% and 7% ±16.7% respectively, Figure 5C.

## Discussion

Malignant tumors frequently silence genes that control cell growth, differentiation and apoptosis through DNA methylation at their promoter regions; consequently, their reactivation by DNA methylation inhibitors has raised considerable interest as anti-tumor therapy. The present focus on DNA methylation has opened a new way to clinically test the ability of diverse compounds as tumor suppressor gene transcriptional reactivators. We previously reported that hydralazine induces *in vitro *and *in vivo*, demethylation and transcriptional reactivation at the mRNA and protein levels of the ER, RARβ and p16 genes [26]. The present study confirms our previous findings in a larger number of patients receiving different hydralazine dosage levels for a 10 day period. Our results demonstrate that hydralazine can demethylate and re-express tumor suppressor genes in previously untreated cervical cancer patients in all the tested dosages. Theses effects are accompanied by no change in global tumor DNA methylation evaluated by two methods, and lack of demethylation in the imprinted gene H19 and the "normally methylated" 1.2 clone in peripheral mononuclear blood cells.

Hydralazine, a widely available peripheral vasodilator agent, has been extensively used for high blood pressure, heart failure and pregnancy-associated hypertensive disorders [32-34]. Hence, its evaluation as a demethylating agent in a clinical trial involving cancer patients could proceed with no major concerns regarding unexpected toxicity and long-term side effects aside of its known capacity to induce a lupus-like condition [35]. Evaluation of tumor DNA demethylating agents in patients bearing solid tumors is troublesome due to the need of repeated tumor sampling. Cervical cancer is easily accessible for repeated tumor sampling and thus we chose to study patients with newly diagnosed cervical cancer. In order to avoid treatment delay in the studied group, we scheduled the present study between the date of diagnosis and the beginning of chemoradiation. In addition, three recent publications have consistently shown a number of tumor suppressor genes to be methylated in primary cervical tumors [36-38]. Our results demonstrate that 70% of the pretreatment samples analyzed had at least one gene methylated, and all 16 patients had at least one methylated gene. The methylation frequency ranged from 3/16 (19%) to 16/16 (100%) for p16 and DAPK genes respectively. (Figure 1A). Despite the fact that our global gene methylation frequency is remarkably similar to the frequencies reported by other authors [36-38] who found 86%, 74% and 79% respectively, we encountered differences in individual gene methylation frequencies which most likely stem from the different patient populations analyzed such as invasive versus pre-invasive disease, tumor histologies, and clinical stages (Table 6). Nevertheless, our results and those obtained in other studies as shown in Table 6, suggest that cervical cancer is a good tumor model to evaluate the effect of DNA demethylating agents upon a small number of individual genes found consistently methylated in high proportion. As expected, there were genes "fully" unmethylated or "fully" methylated but we also observed cases showing both methylated and unmethylated bands. Such ambiguous methylation pattern as evaluated by methylation-specific PCR is commonly observed when patient tumor samples are studied which probably results from certain degree of normal tissue/cell biopsy contamination. This may rise concerns on whether the demethylating effect showed post-treatment is consequence of the treatment and not due tumor heterogeneity, however, in all pretreatment and post-treatment biopsies the major component of the tumor was malignant cells (Figure 3A); On the other hand, if demethylation were the result of chance alone we would expect to have cases in both directions, that is, methylated tumors that demethylated, and demethylated tumors that methylated. The latter never occurred, which strongly supports that the demethylating effect was caused by hydralazine. To gain further insight into the issue of tumor heterogeneity, we multi-sampled a tumor and submitted to methylation analysis each of the tumor fragments containing malignant cells. As shown in Figure 3B, there were no changes in the pattern of methylation in the four separate tumor areas of the tumor. This result strongly supports the observation that demethylation is an effect of hydralazine.

Hydralazine is considered a safe drug with hypertension and hearth failure dosing ranging from 50 mg day to 400 mg/day [33,34]. However, higher doses produce symptomatic side effects mainly derived from its cardiovascular effects. Based on this, we felt important to determine the lowest possible dose that induced DNA demethylation to be used in future clinical trials. In the present study we demonstrate that at a dose range between 50 mg and 150 mg a day for 10 days, hydralazine not only induces gene demethylation in roughly half the evaluated gene/tumors, but we also observed re-expression in two-thirds of the informative cases. However, due to the limited sample size of this study we could not establish whether the effectiveness of this demethylating agent is gene or patient-dependent.

At the present time there is limited information regarding cancer treatment efficacy, demethylating and gene re-expressing profiles of the various DNA demethylating agents in the clinical setting. There are three clinical trial reports evaluating 5-aza-2'-deoxycytidine. Two of these were performed in patients with myelodysplasic syndrome and relapsed leukemia patients. Daskalakis et al., using the quantitative assay Ms-SNuPE found basal hypermethylation of p15 gene in 15/23 patients (65%) which decreased in nine of 12 patients sequentially analyzed after at least a course of low-dose decitabine. As expected, reactivation of the p15 protein expression was found in bone marrow biopsies of four out of eight patients analyzed in this study. Interestingly, response (3 CR) was observed on all nine patients in whom p15 gene demethylation occurred [39]. More recently, a phase I study with escalating doses of decitabine (5, 10, 15 or 20 mg/m^2 ^IV over 1 hour daily, 5 days a week for two consecutive weeks) found responses in 11/18 patients (61%), however, no significant decline in p15 methylation after treatment with decitabine regardless of the response was observed. Moreover, in the three patients that showed >50% demethylation, evaluated by the COBRA assay there was no response. Authors speculated on the poor sensitivity of this technique to explain their results [40]. Evaluation of these nucleoside analogs in solid tumors has also shown inconsistent results. Aparicio et al., reported a phase I study using 5-aza-2'-deoxycytidine in patients with advanced solid tumors using escalating doses of 20, 30, 40 mg/m^2 ^using a 72-hour continuous infusion every 28 days. The quantitative Methyl-Light reaction was used to evaluate changes in promoter methylation in 19 genes but no consistent evidence of gene demethylation was documented despite grade 4 neutropenia was found in almost a third of the patients. This latter finding argues against its use as DNA demethylating agent in solid tumors because despite such toxicity the steady-state levels reached during the study (0.1–0.2 μM) are below the levels needed in vitro to demethylate gene promoters [41]. On the other hand, a number of genes showed increased methylation which could be derived from the cytotoxicity of this nucleoside analog. It is well-known that most of the cytotoxic agents lead to an increase in DNA methylation in vitro and in cancer patients [42].

Among the non-nucleoside demethylating agents, MG98, a DNMT1 antisense oligonucleotide, has been tested in a phase I study to treat solid tumors. In contrast to the studies discussed above no attempt was done to evaluate gene methylation in tumors although DNMT1 mRNA levels were investigated in peripheral blood cells; no consistent decrease was observed [25].

Our results demonstrate that hydralazine is quite promising in regards to its gene demethylating and tumor suppressor gene reactivating activities without significant side effects. It is noteworthy that a dose-effect level in the frequency of demethylation was not observed. A pharmacokinetic correlation analysis would have been useful for the interpretation of these findings, nonetheless, the pharmacokinetics of this drug is relatively well-known and some assumptions can be made. After a single 1 mg/kg oral dose and after the fifth 1 mg/kg dose given every 12 hours, the peak concentrations achieved are in the range of 0.12–1.31 μM and 0.10–1.39 μM respectively and AUC values range between: 4.0–30.4 and 3.2–38.5 μM-minute, respectively [43]. These concentrations were likely achieved in our patients at the doses used which are close to the concentrations needed to achieve gene demethylation and re-expression in vitro [26]. It is not clear why we observed such variable gene demethylation rates irrespective of the doses used, this may be the result of the small number of patients studied as well as the additional variability imposed by the acetylator phenotype which may vary from 30 to 70% according to some studies performed in Latin American Hispanic populations [44].

After years characterizing the role of DNA methylation in cancer, it has become clear that both hypermethylation and hypomethylation are by their own cancer causative or at least cancer promoting, in animal models. This has led to consider the use of demethylating agents for cancer treatment a two-edge sword [45]. Such consideration is debatable as the profound DNA demethylation levels achieved in animal models can be hardly pharmacologically reproduced in patients. The present study demonstrates that hydralazine at the doses used not only does not demethylate genes which are "normally methylated" such as H19 and clone 2.1 but also fails to produce global DNA demethylation as evaluated by capillary electrophoresis which is a powerful and accurate method to quantitate methylated and unmethylated cytosines [46], and by cytosine extension assay which is also a sensitive method to underscore abnormal methylation patterns [31]. We can only speculate on the nature of these findings. At the individual gene level, a plausible explanation could be that certain "normally methylated" genes could lie within a *milieu *prone to rapid re-methylation and/or that certain genes have higher "availability" of the elements that compose the methylation machinery, therefore the demethylating agent would fail to produce demethylation of these sequences at least at a detectable levels by the current methods of analysis. This view is supported by the reporting that in mice 5-azacytidine does not change the methylation status of H19 [47]. In regard to global DNA methylation changes most likely is just a matter of dose as we have in vitro evidence that hydralazine produces global DNA hypomethylation in MCF-7 cells as evaluated by the same methods here used. Alternatively, changes in global ^m^C content induced by hydralazine are not sufficient to be detected by the methods we used.

## Conclusion

In conclusion, hydralazine used at standard doses for the treatment of cardiovascular conditions is an effective demethylating and tumor suppressor gene transcriptional reactivator causing no decrease in DNA ^m^C content for solid tumors. Our results are in agreement with those reported by Chan et al., who found substantial degrees of demethylation at all latent and lytic Epstein-Barr virus promoters examined by methylation-specific PCR in nasopharyngeal cancer patients treated with 5-azacytidine [48]. A phase II clinical study using hydralazine in combination with standard cytotoxic chemotherapy is being planned as proof of concept that the reactivation of tumor suppressor genes silenced by DNA methylation increases chemotherapy efficacy in solid tumors.

## Competing interests

The author(s) declare that they have no competing interests.

## Authors' contributions

P Z, B S-P, E P-C, C T-B, L TC, and A C-B performed the methylation analysis and gene expresión experiments; LC cared for the patients; AR-V and KS performed the capillary electrophoresis analysis; EA and GC critically read and contributed to the discussion; J C-V, performed the pathological analysis; and A D-G conceived the study and wrote the manuscript.

## Pre-publication history

The pre-publication history for this paper can be accessed here:



## Figures and Tables

**Table 1:** Primers and conditions used for MSP.

Primer set	Sense 5'-- 3'	Antisense 5'--- 3'	Size	Annealing
p16 M	TTATTAGAGGGTGGGGCGGATCGC	GACCCCGAACCGCGACCGTAA	150	62°C
p16 U	TTATTAGAGGGTGGGGTGGATTGT	CAACCCCAAACCACAACCATAA	151	62°C
RARβM	TCGAGAACGCGAGCGATTCG	GACCAATCCAACCGAAACGA	146	55.5°C
RARβU	TTGAGAATGTGAGTGATTTGA	AACCAATCCAACCAAAACAA	146	55.5°C
MGMT M	TTTCGACGTTCGTAGGTTTTCGC	AACCAATCCAACCAAAACAA	81	55.7°C
MGMT U	TTTGTGTTTTGATGTTTGTAGGTTT	AACTCCACACTCTTCCAAAAAC	93	55.7°C
FHIT M	TTGGGGCGCGGGTTTGGGTTTTTA	CGTAAACGACGCCGACCCCACTA	74	55.5°C
FHIT U	TTGGGGTGTGGGTTTGGGTTTTTA	CATAAACAACACCAACCCCACTA	74	55.5°C
DAPK M	GGATAGTCGGATCGAGTTAACGT	CCCTCCCAAACGCCGA	98	55.7°C
DAPK U	GGAGGATAGTTGGATTGAGTTAAT	CAAATCCCTCCCAAACACCAA	106	55.7°C
APC M	TATTGCGGAGTGCGGGTC	TCGACGAACTCCCGACGA	100	56°C
APC U	GTGTTTTATTGTGGAGTGTGGGTT	CCAATCAACAAACTCCCAACAA	110	56°C
GSTp1 M	TTCGGGGTGTAGCGGTGGTC	GCCCCAATACTAAATCACGACG	91	56°C
GSTp1 U	GATGTTTGGGGTGTAGTGGTTGTT	CCACCCCAATACTAAATCACAACA	97	56°C
H19 M	TTATAAAATCGAAAATTACGCGCGA	AGATGATTTTCGTGAATTTTGCG	136	55°C
H19 U	TATAATTATAAAATCAAAAATTACA	TTTTAGATGATTTTTGTGAATTTT	145	55°C
ER M	CGGTTGGAGTTTTTGAATCGTTC	CTAGCGTTAACGACGACCG	151	55°C
ER U	ATGAGTTGGAGTTTTTGAATTGTTT	ATAAACCTACACATTAACAACAACCA	158	55°C
Clone 1.2 M	ATGAGTTGGAGTTTTTGAATTGTTT	AATAATAAACGTAACGCCCGCGAAC	258	63°C
Clone 1.2 U	GGTTGTTTGGTTTTTATTGGGATGTTTTT	CCTAAATAATAAACATAACACCCACAAAC	258	63°C

**Table 2:** Primers and conditions used for RT-PCR

Primer set	Sense 5'-- 3'	Antisense 5'--- 3'	Size	Annealing
p16	AGCCTTCGGCTGACTGGCTGG	CTGCCCATCATCATGACCTGG	150	60°C
RARβ	GACTGTATGGATGTTCTGTCAG	ATTTGTCCTGGCAGACGAAGCA	146	50°C
MGMT	GCTGCAGACCAVCTCTGTGGCACG	GCCGCCTCTTCACCATCCCG	81	50°C
FHIT	ATGTCGTTCAGATTTGGCCAAC	TCATAGATGCTGTCATTCCTGT	340	53°C
DAPK	AACCCATCATCCATGCCATC	TCTCTCCTTCTCGGTTCTTGA	200	51°C
APC	GAGACAGAATGGAGGTGCTGC	GTAAGATGATTGGAATTATCTTCT	180	56°C
GSTp1	TCCGCTGCAAATACATCTCC	TGTTTCCCGTTGCCATTGAT	320	50°C
ER	GGAGACATGAGAGCTGCCAAC	CCAGCAGCATGTCGAAGATC	480	55°C

**Table 3:** Clinical characteristics of patients

Number	16
Mean Age	51.8 (35–75 years)
Histology	
Squamous	16 (100%)
FIGO Stage*	
IIB	8 (50%)
IIIB	8 (50%)
Performance Status**	
0	10 (62%)
1	6 (38%)

*International Federation of Gynecology and Obstetrics,

** World Health Organization Criteria

**Table 4:** Toxicity to hydralazine expressed by number of patients suffering the event (16 patients)

Toxicity	4 Patients in each dose level (mg			
	50	75	100	150
Nausea	2/4	-	2/4*	2/2
Vomiting	-	-	-	-
Dizziness	-	1/4*	1/4	-
Fatigue	2/4	2/4	3/4	1/4
Flushes	-	-	-	-
Headache	2/4	1/4	3/4	2/4
Edema	-	-	-	-
Palpitations	1/4	2/4	2/4	3/4

* Grade 2 toxicity. All other were grade 1.

**Table 5:** Methylation and gene re-expression of the 12 informative cases.

		Pre-treat		Post-treat		Re-expression
Gene	dose level	M	U	M	U	Yes/No

DAPK	50	+	-	+	-	No
DAPK	50	+	-	+	-	No
GSTp1	50	+	-	+	-	No
FHIT	100	+	-	+	+	Yes
FHIT	150	+	-	+	+	Yes
MGMT	75	+	-	+	+	Yes
MGMT	150	+	-	+	+	Yes
GSTp1	50	+	-	+	+	Yes
DAPK	75	+	-	-	+	Yes
ER	75	+	-	-	+	Yes
GSTp1	150	+	-	-	+	Yes
GSTp1	75	+	+	-	+	Yes

**Table 6:** Frecuency of promoter methylation of individual genes (%).

	APC	ER	FHIT	GSTp1	MGMT	P16	RARB	DAPK	ANY
This study	94	25	88	88	81	19	62	100	70
Narayan (ref 35)	11	-	11	-	5	8.5	29	45	86
Virmani (ref 34)	-	-	32	21	26	42	26	-	74
Dong (ref 36)	32	-	-	-	8	30	-	51	79
